# Alcohol-Induced Molecular Dysregulation in Human Embryonic Stem Cell-Derived Neural Precursor Cells

**DOI:** 10.1371/journal.pone.0163812

**Published:** 2016-09-28

**Authors:** Yi Young Kim, Ivan Roubal, Youn Soo Lee, Jin Seok Kim, Michael Hoang, Nathan Mathiyakom, Yong Kim

**Affiliations:** 1 Laboratory of Stem Cell & Cancer Epigenetic Research, School of Dentistry, University of California Los Angeles, 10833 Le Conte Avenue, 73–041 CHS, Los Angeles, CA, 90095, United States of America; 2 Center for Oral and Head/Neck Oncology Research Center, Division of Oral Biology & Medicine, UCLA School of Dentistry, 10833 Le Conte Avenue, 73–022 CHS, Los Angeles, CA, 90095, United States of America; 3 UCLA’s Jonsson Comprehensive Cancer Center, 8–684 Factor Building, Box 951781, Los Angeles, CA, 90095, United States of America; 4 UCLA Broad Stem Cell Research Center, Box 957357, Los Angeles, CA, 90095, United States of America; University of Texas at Austin Dell Medical School, UNITED STATES

## Abstract

Adverse effect of alcohol on neural function has been well documented. Especially, the teratogenic effect of alcohol on neurodevelopment during embryogenesis has been demonstrated in various models, which could be a pathologic basis for fetal alcohol spectrum disorders (FASDs). While the developmental defects from alcohol abuse during gestation have been described, the specific mechanisms by which alcohol mediates these injuries have yet to be determined. Recent studies have shown that alcohol has significant effect on molecular and cellular regulatory mechanisms in embryonic stem cell (ESC) differentiation including genes involved in neural development. To test our hypothesis that alcohol induces molecular alterations during neural differentiation we have derived neural precursor cells from pluripotent human ESCs in the presence or absence of ethanol treatment. Genome-wide transcriptomic profiling identified molecular alterations induced by ethanol exposure during neural differentiation of hESCs into neural rosettes and neural precursor cell populations. The Database for Annotation, Visualization and Integrated Discovery (DAVID) functional analysis on significantly altered genes showed potential ethanol’s effect on JAK-STAT signaling pathway, neuroactive ligand-receptor interaction, Toll-like receptor (TLR) signaling pathway, cytokine-cytokine receptor interaction and regulation of autophagy. We have further quantitatively verified ethanol-induced alterations of selected candidate genes. Among verified genes we further examined the expression of *P2RX3*, which is associated with nociception, a peripheral pain response. We found ethanol significantly reduced the level of *P2RX3* in undifferentiated hESCs, but induced the level of *P2RX3* mRNA and protein in hESC-derived NPCs. Our result suggests ethanol-induced dysregulation of *P2RX3* along with alterations in molecules involved in neural activity such as neuroactive ligand-receptor interaction may be a molecular event associated with alcohol-related peripheral neuropathy of an enhanced nociceptive response.

## Introduction

Alcohol consumption is recognized as the leading preventable cause of birth defects and mental retardation. High levels of alcohol consumption during pregnancy can result in fetal alcohol spectrum disorders (FASDs), which is characterized by prenatal and postnatal growth restriction, craniofacial dysmorphology and structural abnormalities of the central nervous system [1]. Depending on conditions and manifestations, these damages are referred as fetal alcohol syndrome (FAS), alcohol-related birth defects (ARBDs), and alcohol-related neurodevelopmental disorder (ARND). While the developmental defects from alcohol abuse during gestation have been described, it is still unanswered about what are the specific mechanisms by which alcohol mediates these injuries [2, 3]. This is important question to address to identify affected children at an early age and intervene to prevent or mitigate the damage.

The effect of alcohol on development has been widely studied in many different animal species [4]. Adverse effect of alcohol on brain function has been well documented. Especially, the teratogenic effect of alcohol on neurodevelopment during embryogenesis has been demonstrated in animal models, which could be a pathologic basis for FASDs [1, 3]. It has been demonstrated that alcohol exposure during preimplantation period has significant effect on embryo development [5]. Reports have demonstrated genetic, cellular, and biochemical association of alcohol with teratogenesis [6–9]. The wide range of physiological and morphological defects associated with in utero alcohol exposure suggest that the etiology of FASDs involve a high degree of cellular and molecular heterogeneity. Gastrulation period is considered to be the most sensitive to teratogenic insult, suggesting that differentiating cells might be especially vulnerable to the teratogenic effects of alcohol [7].

Currently, it is not clearly established what causes FASDs. Recently, epigenetic regulations have emerged as potential mechanisms associated with alcohol teratogenesis. Epigenetic imprinting or genome-wide epigenetic reprogramming has been proposed as a mechanism responsible for alcohol-induced teratogenesis in preimplantation embryos [2, 3]. Interestingly, even paternal or maternal alcohol consumption prior to conception has been shown to result in a wide range of birth defects and fetal abnormalities. It is likely that alcohol-induced epigenetic changes in the gametes or within germ line are responsible for pre-conceptional effects of alcohol [10]. Considering the importance of epigenetic factors in development, especially in central nervous system development and dysfunction, it is quite reasonable to link epigenetic mechanisms as potential regulatory events involved in alcohol teratogenesis [2, 11–13].

Embryonic stem cells (ESCs) are pluripotent cells that can be derived into all lineages of cells in the organism [14]. Due to this biological competency of ESCs, beneficial utility of ESCs for regenerative medicine has been suggested in many applications [15]. In addition, ESC has been proven to be a useful tool to study mechanisms associated with the pathogenesis of genetic disorders, especially disease-associated molecular alterations at the early stage of fetal development [16]. ESCs provide us with an opportunity to establish an experimental model to study the functional effects of genetic alterations on normal embryo development and further to test tools to intervene deleterious effects of genetic alterations on the later stage of life. Stem cell models are beneficial to developmental studies especially where *in vivo* molecular/cellular study models are not available.

Stem cells are especially vulnerable to ethanol (EtOH) toxicity through decreases in pluripotency, survival capacity, and/or altered differentiation [7]. Studies have shown that alcohol has significant effect on molecular and cellular regulatory mechanisms in ESC differentiation [17]. More interestingly, it has been demonstrated that alcohol induces alteration in genes involved in neural development in ESCs [18, 19]. It is known that gastrulation periods of ESCs including neuronal differentiation process require epigenetic controls, especially DNA methylation [2]. Our recent studies have described the molecular signatures of EtOH’s effects on stem cell potency and differentiation in human embryonic stem cells (hESCs) [20]. Studies also showed that EtOH exposure reduces neuronal stem cell numbers in developing and adult brains [21, 22].

As a surrogate model for adult neural stem cells, pluripotent hESCs can be differentiated into neural progeny and used to elucidate mechanisms underlying early human neurogenesis. An established *in vitro* neural stem cell model will be useful for the evaluation of developmental regulatory mechanisms and also the significance of their alterations in pathological conditions such as developmental disorders or exposure to neuroteratogens [19]. We have derived neural stem cells from human embryonic stem cells as a model to study the effect of teratogen in neural development. We have successfully derived neural precursor cells exhibiting key molecular features of neural stem cells, which will be useful for experimental application [23]. Our established model has further been used to assess the molecular effect of alcohol treatment on neural stem cell population. It was found that alcohol exposure of neural precursor cells resulted in significant alterations of molecular signatures that may have functional role in neural stem cell maintenance and development and may be further involved in deleterious cellular effect of alcohol exposure.

## Materials and Methods

### Human embryonic stem cell culture and derivation of neural stem cells

**Fig 1:**
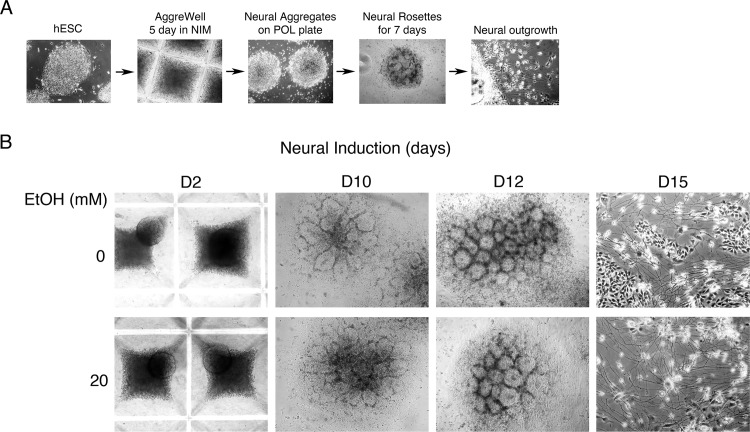
Neural differentiation of human embryonic stem cells *in vitro*. **A**. Neural differentiation of hESC *in vitro*. Human embryonic stem cells were subjected to embryoid body (EB) formation using AggreWell for 5 days in neural induction medium (NIM). Neural aggregates were seeded on plates coated with poly-L-ornithine/laminin (PLO/L) and cultured with NIM for additional 7 days to develop neural rosette structure. After 7 days, the neural rosettes were dislodged and then replated for the expansion of neural precursor cells for 3–5 days. **B**. Human embryonic stem cells were subjected to embryoid body formation using AggreWell for 5 days in neural induction medium. Neural aggregates were seeded on poly-L-ornithine/laminin coated plates and cultured with NIM for 7 days to develop neural rosette structure. Ethanol treatment was initiated a day after plating the neural aggregates onto PLO/L plates. For ethanol treatment cells were fed with fresh medium every day by alternating a treatment with 20 mM ethanol for 1 day and a withdrawal for 1 day. Treatment was continued till the end of neural expansion. After 7 days, the neural rosettes were dislodged and then re-plated for the expansion of neural precursor cells for 5 days.

Human embryonic stem cells (H1 and H9 lines) obtained through license agreement with WiCell Research Institute (Madison, WI) were cultured on mouse embryonic fibroblast (MEF) feeder layer and were transferred to mTeSR1 serum free human embryonic stem cell (hESC) culture system (STEMCELL Technologies Inc., Vancouver, Canada). Neural differentiation of hESCs was performed by using STEMdiff Neural System (STEMCELL Technologies Inc., Vancouver, Canada) according to the manufacturer’s instruction as described in our previous publications [23, 24]. After 7 day differentiation, morphological assessment and scoring of neural rosettes were done to ensure 50% or more of the area of each aggregate was filled with neural rosettes (as shown in Fig 1). On day 7, neural rosettes were selected away from contaminating flat cells and collected. The rosettes were resuspended in pre-warmed NIM and cultured on 6-well plates precoated with poly-L-Ornithine and laminin (PLO/L) with daily full medium changes using pre-warmed STEMdiff NIM (without or with 20 mM ethanol) for 5 days with alternating treatment for a day and withdrawal for a day. Cells were EtOH concentration was chosen for its physiological relevance in that 20 mM is equivalent to DUI level and 50 mM falls within levels measured in alcoholics [25].

### Immunofluorescence analysis

To ensure proper neural differentiation of hESCs, the same experimental procedure was applied to a set of cells plated on the coverslips. The level of neural markers (Nestin, Sox2, Musashi and beta-3 tubulin) was assessed by immunofluorescence microscopy. For IF analysis cells were seeded on a coverslip coated with Matrigel in a 6 well plate. The cells were fixed with 4% paraformaldehyde/PBS for 30 min at room temperature and washed with PBS. IF analysis for neural stem cell markers was done by using Human Neural Stem Cell Characterization Kit (EMD Millipore, Billerica, MA). Samples were incubated with blocking solution (5% normal donkey serum, 0.3% Triton X-100 in 1X PBS) for 30 min at room temperature. After washing three times (5 min each) with 1X PBS, samples were incubated with primary antibody [mouse anti-Nestin (1:500), rabbit anti-Sox2 (1:1000), mouse anti-βIII tubulin (1:1000), or rabbit anti-Musashi1 (1:500)] diluted in 1X PBS (or blocking solution) and incubated overnight at 4°C. As a negative control, we have included the same isotype rabbit IgG or mouse IgG at equivalent concentrations. After washing three times for 5 min each with 1X PBS, samples were incubated with appropriate secondary antibody [donkey anti-rabbit IgG-FITC (1:500) or donkey anti-mouse IgG-rhodamine (1:500)] for 2 hrs at RT. After washing, the slips were mounted on the slide with mounting medium containing DAPI (Vector Labs, Inc., Burlingame, CA) and fluorescence imaging was performed with Olympus IX81 imaging microscope.

### RNA isolation and microarray analysis

RNA isolation and microarray analysis was done as described [20, 24]. Briefly, the neural aggregates at D10 for the formation of rosettes and at D15 (5 days after re-plating the rosette clusters for the expansion of neural precursor cells) were used for total RNA isolation by using RNeasy mini kit (Qiagen, Valencia, CA). RNA purity and concentration was determined by NanoDrop, ND-1000 spectrophotometer (Thermo Scientific, Indianapolis, IN) and microfluidics-based platform 2100 Bioanalyzer (Agilent Technologies, Santa Clara, CA). Samples in biological duplicate were hybridized to Affymetrix Human Genome Plus 2.0 (Cat. No. 900469). Standard quality control metrics recommended by Affymetrix including image quality, signal distribution and pair wise scatter plots were used for all arrays. Mas5.CHP files were generated for each array by MAS 5.0 (Affymetrix, Santa Clara, CA) and combined to a final RESULTS.MAS5.TXT file [20, 24].

### Quantitative RT-PCR analysis

For validation of gene expression by quantitative RT-PCR analysis was performed as describe previously [20]. Total RNA was first subjected to DNase digestion with Turbo DNA-free kit (Life Technologies, Grand Island, NY). 2 μg of total RNA treated with DNase was used to synthesize cDNA by using iScript cDNA synthesis kit (Bio-Rad, Hercules, CA) in 40 μl reaction mixture. The resulting cDNA was diluted (mixed with H_2_O by 1:4) and 1.5 μl of diluted cDNA was used per well (in 10 μl reaction volume) in a 384 well plate using LightCycler 480 SYBR Green I master mix (Roche Diagnostics, Indianapolis, IN).

## Results

### Derivation of neural precursor cells from human embryonic stem cells

**Fig 2:**
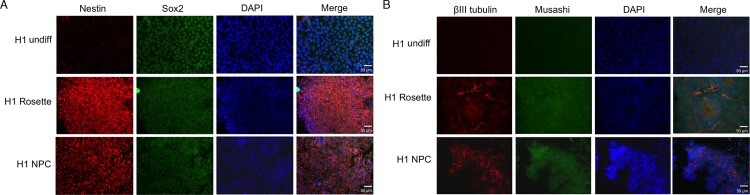
Immunofluorescence analysis to confirm neural differentiation. Induced expression of neural markers in hESC-derived neural stem cells. Neural differentiation of hESCs was confirmed by examining the expression of neural markers. **A**. Nestin and SOX7 and **B**. beta-tubulin and Musashi expressions were noticeably increased in rosettes and NPCs. SOX2 expression remained high in pluripotent hESCs and also hESC-derived neural lineage cells. Cells were counterstained with DAPI for nuclei (scale bar, 50 μm).

To establish a model system to examine the molecular effects of EtOH on neural differentiation and maintenance of neural stem cells, we used a commercial culture system developed for neural differentiation of human embryonic stem cells (STEMCELL Technologies Inc., Vancouver, Canada) as we have previously reported [23, 24]. Human embryonic stem cells (hESCs) exponentially growing on mouse embryonic fibroblast (MEF) feeder were first adapted to the feeder-free culture system using mTeSR1 medium (STEMCELL Technologies Inc., Vancouver, Canada) and subjected to embryoid body formation in neural differentiation medium as described in Materials and Methods. Fig 1A shows the overall scheme for the derivation of neural precursor cells from hESCs. Neural differentiation was performed in the presence or absence of 20 mM EtOH (Fig 1B). Cells were collected for RNA purification at day 12 (rosette structure) and day 15 (neural precursor cells). Morphological observation clearly showed the formation or rosette structures and neural differentiation (Fig 1B). However, EtOH’s effect on the process could not be quantitatively assessed by microscopic observation. To examine if hESCs were properly specified into neural cells the expression of neural lineage markers was assessed by immunofluorescence microscopy (Fig 2). Compared to undifferentiated hESCs, rosette and NPC showed induced expression of neural maker Nestin, neuron-specific class III beta-tubulin (TBB3 or Tuj1) and Musashi (Fig 2). Expression of stemness factor Sox2 was maintained in undifferentiated, rosette and NPC as expected (Fig 2A). Even though immunofluorescence microscopy analysis was not proper to quantitatively compare the expression level of markers, it seems that EtOH may have inhibitory effect on the expression of Nestin and Musashi, notably in rosette structure (Fig 2A).

To further quantitatively characterize the neural specification of hESCs into rosette and NPCs, we have performed quantitative RT-PCR analysis on neural markers (S1 Fig). We found significant induction of Nestin, Musashi and Tuj1 mRNA in neural rosettes and NPCs (S1A Fig). Our analysis showed that 20 mM EtOH treatment resulted in significant reduction of Nestin and Musashi mRNA (S1B Fig). But this reduction seems marginal and may not cause dramatic decrease in the steady state level of mRNA’s considering highly induced levels of these mRNA’s after neural differentiation, which may have marginal effect on the level of corresponding protein as shown in Fig 2. We have previously demonstrated that EtOH treatment resulted in significant reduction of pluripotent markers such as Oct4 and Sox2 in undifferentiated hESCs [20]. It is known that Sox2 plays a role in the neural specification and proliferation of NPCs while Oct4 and Nanog is involved in mesendoderm differentiation [26]. We found that neural differentiation resulted in dramatic downregulation of Oct4 mRNA, but the level of Sox2 was comparably maintained and was not greatly affected by EtOH treatment in rosettes and NPCs (S1C Fig).

### Profiling of gene signatures affected by ethanol treatment during differentiation of hESCs to neural precursors

We have used our hESC neural differentiation model to profile gene signatures significantly altered by alcohol exposure, specifically during directed differentiation into neural precursors *in vitro*. H1 and H9 hESCs were independently directed to neuronal differentiation in the presence or absence of 20 mM ethanol in biological duplicates. Mixed population of cells pooled from each replicate was harvested at day 12 for neural rosette cells and at day 15 for neural precursor cells (NPC). Gene expression microarray analysis was done using samples from H1 hESC line. Total RNA was prepared and subjected to gene expression microarray as described in Materials and Methods by using Affymetrix Human U133A 2.0 arrays (data accession number GSE56906). We have analyzed the processed data by Weighted Gene Correlation Network Analysis (WGCNA) and also by simple fold change comparisons against control samples.

**Fig 3:**
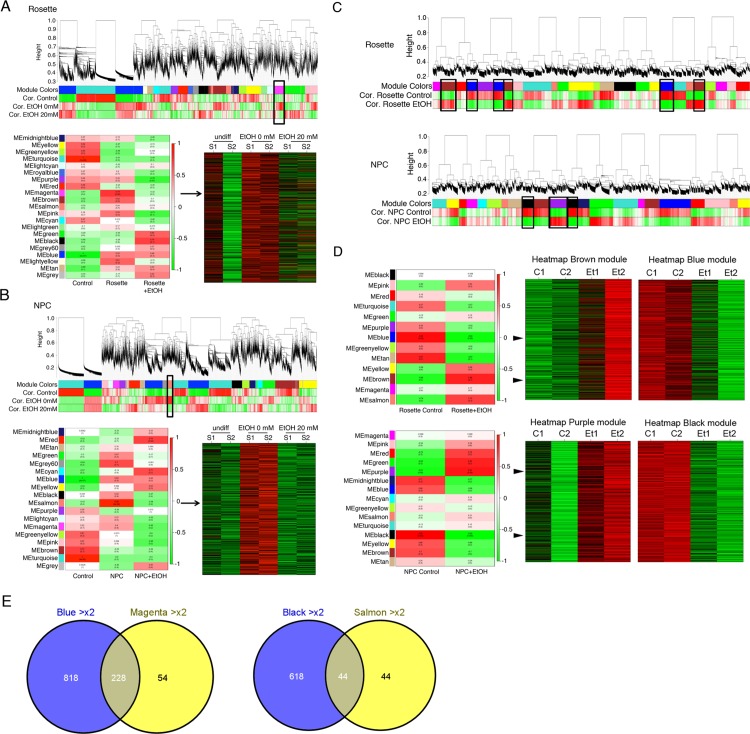
Gene expression analysis and WGCNA. WGCNA analysis on EtOH-induced transcriptomic alterations in neural rosettes and NPC populations derived from hESCs compared to undifferentiated parental hESCs. **A**. Result from rosettes identified the magenta module of genes that were upregulated in rosettes, but downregulated by EtOH treatment. **B**. Analysis for NPCs showed the salmon module of genes that were upregulated in NPCs, but downregulated by EtOH treatment. Heatmaps are shown for modules with most significant association with EtOH treatment identified from the module trait map of the module eigengene (ME) from two biological duplicates (S1 and S2) to each treatment. Rosettes showed less extent of association than NPCs. **C**. In addition, we have performed WGCNA analysis on gene expression microarray data to directly assess EtOH’s effect on transcriptomic alterations in neural rosettes and NPCs with and without EtOH treatment. **D**. Heatmaps are shown for modules with most significant association upon EtOH treatment (biological duplicate of Et1 and Et2) compared to without EtOH (biological duplicate of C1 and C2). The Brown module (upregulated- red) and the blue module (downregulated- green) with EtOH treatment in neural rosettes and the purple module (upregulated- red) and the black module (downregulated- green) upon EtOH exposure in NPCs. **E**. Common genes that demonstrated consistent alterations were identified. For example, genes in the blue module (downregulated >2 fold in EtOH-treated rosettes compared to untreated rosettes) were compared to the magenta module (>2 fold upregulated during differentiation into rosettes and then downregulated >2 fold upon EtOH treatment). Similar analysis was done with the black and salmon modules to identify genes truly downregulated in NPC with EtOH treatment.

We have initially performed WGCNA on datasets from both rosette and NPC samples altogether and found that the effects of differentiation (hESCs to neural rosette and further to NPC) were too overwhelming to see the effect of EtOH treatment on gene signatures (data not shown). Correlation of gene expression to EtOH treatment was not strong enough over the effect of differentiation (data not shown). Therefore, we decided to analyze the dataset for the effect of EtOH on rosette cells separately from on NPC. We performed WGCNA on undifferentiated hESCs (undiff), differentiated without EtOH treatment (EtOH 0 mM) and differentiated with EtOH treatment (EtOH 20 mM). We examined correlations to differentiation and EtOH treatment in neural rosette (Fig 3A) or NPC (Fig 3B) from the biological duplicate samples (S1 and S2). We identified modules that are altered by differentiation or altered by EtOH treatment during differentiation. As an example, we have shown in Fig 3 the most significantly associated module with EtOH treatment in rosettes and NPCs. Fig 3A shows the magenta module of genes that were upregulated in rosettes, but downregulated by EtOH treatment. Fig 3B shows the salmon module of genes that were upregulated in NPCs, but downregulated by EtOH treatment. This analysis allowed us to identify gene signatures that were differentially regulated during differentiation into neural rosette or NPC and affected by EtOH treatment.

It was noted that correlation to EtOH treatment was not strong and only a very limited number of modules showed EtOH-dependent association. We reasoned that this was due to the strong effect of neural differentiation on the gene regulation in our model. Next we examined correlative gene expression simply associated with EtOH treatment in differentiated cells. We used datasets from differentiated rosette cells or NPCs with or without EtOH treatment (Fig 3C and 3D). WGCNA on rosette cells (control vs. EtOH treated) showed that the brown module was most significantly correlated to genes upregulated and the blue module was most significantly correlated to genes downregulated upon EtOH treatment (Fig 3C and 3D). A heatmap for each representative module is shown. Similar analysis was done with dataset from NPC cells. The purple module was most significantly correlated to genes upregulated upon EtOH treatment and the black was most significantly correlated to genes downregulated upon EtOH treatment (Fig 3C and 3D). To examine concordance between these two different analysis settings (Fig 3A/3B vs. Fig 3C/3D), we examined genes that were coherently altered by EtOH treatment (Fig 3E). The magenta module (Fig 3A) and the blue module (Fig 3D) represent genes downregulated in rosette cells upon EtOH exposure. Likewise, the salmon module (Fig 3B) and the black module (Fig 3D) represent genes downregulated in NPCs upon treatment with EtOH. The result showed list of genes that were similarly affected by EtOH treatment in two analysis settings.

**Fig 4:**
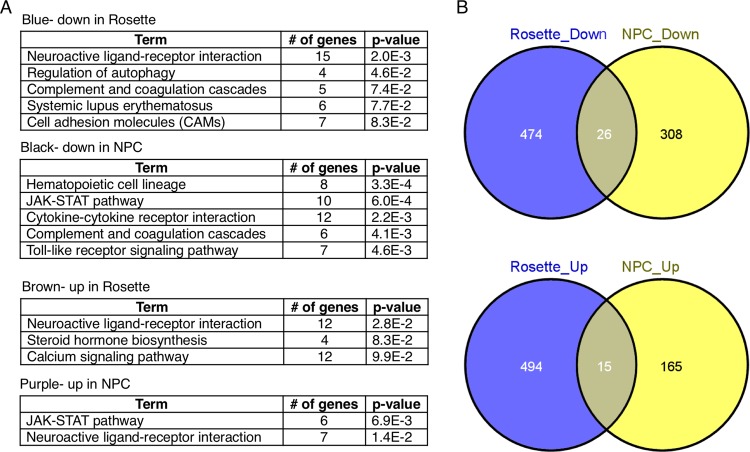
DAVID function analysis of identified genes. **A**. DAVID functional analysis was performed to examine potential functional association of identified genes. Genes in the four major modules (black and purple modules from NPC model and blue and brown modules from neural rosette model) were analyzed. It was found that genes dysregulated upon EtOH treatment in both NPCs and neural rosettes are significantly associated with neuroactive ligand-receptor interaction, JAK-STAT pathway and cytokine-cytokine receptor interaction and TLR signaling pathway. **B**. Genes that are commonly affected by EtOH (greater than 3-fold) in neural rosette and NPC were identified by Venny plot. List of genes are shown in Table 1.

To examine potential effects of EtOH on biological process, we have performed DAVID (The Database for Annotation, Visualization and Integrated Discovery) on genes from the blue (downregulated in neural rosette), the black (downregulated in NPC), the brown (upregulated in neural rosette) and the purple (upregulated in NPC). EtOH treatment in neural rosettes showed alterations of genes potentially involved in neuroactive ligand-receptor interaction, cell adhesion molecules and calcium signaling pathway (Fig 4A). The black module of downregulated genes in NPC upon EtOH treatment was associated with several important signaling pathways, such as JAK-STAT, cytokine-cytokine receptor interaction and Toll-like receptor (TLR) signaling (Fig 4A). On the other hand, the purple module of upregulated genes showed association with neuroactive ligand-receptor interaction (Fig 4A). To examine if there is any concordance in molecular networks in NPCs and neural rosettes affected by EtOH treatment, we have identified common genes in rosettes and NPCs that showed greater than 3-fold changes upon exposure to EtOH by using Venny 2.0.2 (Computational Genomics Service) (Fig 4B). We identified core genes that are similarly regulated by EtOH treatment in rosette cells and NPCs by combining the genes in the blue and the black module for commonly downregulated genes (>3-fold) and by combining the genes in the brown and the purple module for commonly upregulated genes (>3-fold). List of genes are shown in Table 1.

**Table 1:** List of genes that are commonly dysregulated in NPC and neural rosette after EtOH treatment.

Down >3 NPC and Rosette	Up >3 NPC and Rosette
C11orf21	TSHR
C19orf23	ALS2CR11
CD36	DDO
CD84	DOC2B
CLCN1	DOCK4
CNGB3	FKSG83
COL4A3	KCNE3
FLJ32255	KLF14
GPR84	LIN9
HNRNPD	MAP1LC3B2
IFNA2	OPRM1
ITPRIPL2	RHAG
LOC100506517	SCARA5
LOC100507226	TNP2
LOC253039	UPB1
LOC283079	
LOC283674	
MACC1	
NBLA00301	
NPAS3	
OFCC1	
PCBD2	
TLR3	
TNS1	
TRD@	
ZAN	

### Validation of candidate genes differentially regulated by EtOH treatment in NPCs

**Fig 5:**
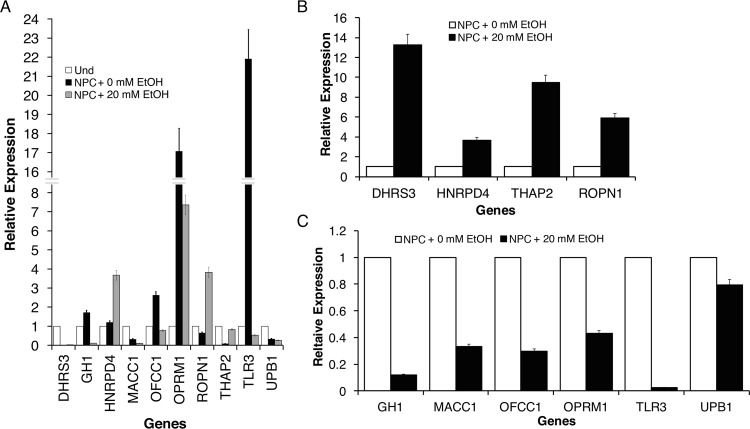
Identification of candidates and validation. qRT-PCR validation of candidate genes that are potentially regulated by EtOH treatment during neural differentiation of hESCs. Some of validation analysis results are shown. From WGCNA we have identified genes that are significantly affected by EtOH treatment in neural stem cells. **A**. Top candidate genes identified showed the effect of EtOH on gene levels in validation analysis by comparing undifferentiated hESCs, untreated NPCs and EtOH treated NPCs. Some of validation result by comparing the level of genes in NPCs with and without EtOH treatment showed **B**. upregulation and **C**. downregulation upon EtOH treatment. We have identified *DHRS3*, *MACC1*, *THAP2*, *TLR3* and *DRD4* are most significantly and consistently validated genes among candidates. Bars are mean ± SEM from triplicates; significant (*p*<0.05) difference from control (one-way ANOVA).

Candidate genes potentially regulated by EtOH treatment during neural differentiation were validated by qRT-PCR analysis (Fig 5). Selected genes were first validated in undifferentiated hESC, NPC with 0 mM EtOH and NPC with 20 mM EtOH. As shown in Fig 5A, we observed significant alterations associated with neural differentiation (neural diff + 0 mM EtOH) compared to undifferentiated control. To better examine the effect of EtOH on gene regulation, NPCs were treated with 20 mM EtOH and relative expression levels of candidate genes were compared to their respective controls (0 mM EtOH) (Fig 5B and 5C). Genes *DHRS3*, *HNRPD4*, *THAP2* and *ROPN1* demonstrated alcohol induced upregulated expression (Fig 5B). By contrast, downregulated expression was seen in the EtOH treatment of *GH1*, *MACC1*, *OFCC1*, *OPRM1*, *TLR3* and *UPB1* (Fig 5C).

**Fig 6:**
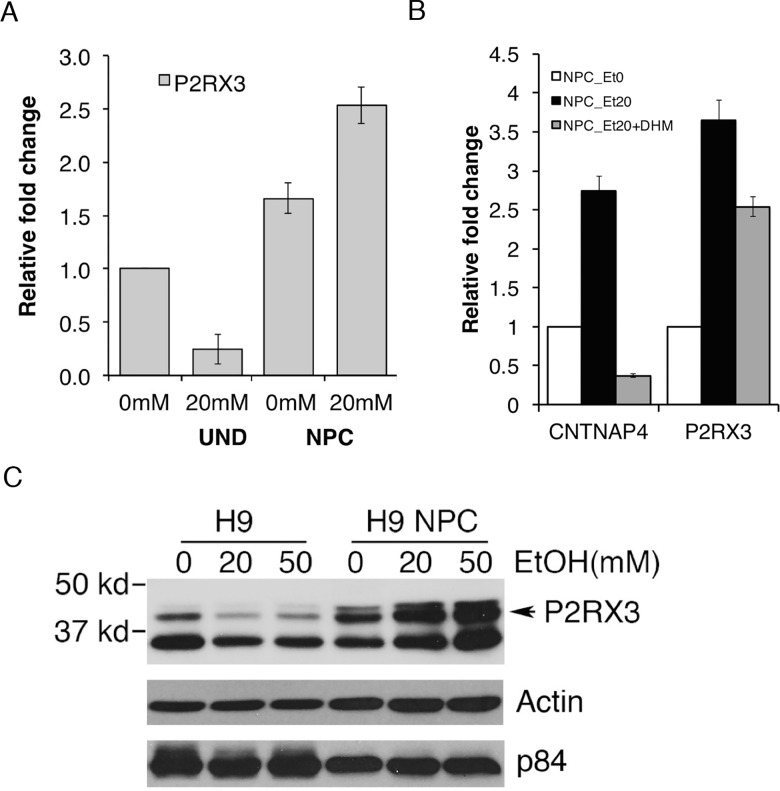
Ethanol induced-alteration of *P2RX3* in undifferentiated hESCs and NPCs. **A.** Comparison of fold changes of *P2RX3* expression in undifferentiated H1 hESCs and hESC-derived NPCs treated with EtOH. *P2RX3* in undifferentiated hESCs was downregulated and conversely upregulated in NPCs with increased EtOH concentration. Bars are mean ± SEM from triplicates; significant (*p*<0.05) difference from control (one-way ANOVA). **B**. The level of *CNTNAP4* and *P2RX3* in H1 NPC was induced by treatment with 20 mM EtOH. Co-treatment with 1 μM dihydromyricetin (DHM), a flavonoid component of herbal medicines that counters acute EtOH intoxication, significantly interfered the effect of EtOH, demonstrating the specificity of EtOH’s effect on the gene expression. Bars are mean ± SEM from triplicates; significant (*p*<0.05) difference from control (one-way ANOVA). **C**. Relative protein levels of *P2RX3* in undifferentiatied H9 hESCs and H9 NPC cell lines treated with 0, 20, and 50 mM EtOH for 48 hrs. From Western analysis, we have confirmed alcohol-induced downregulation of P2RX3 in undifferentiated hESCs and upregulation in NPCs.

The effect of EtOH on the expression of one of the candidate genes, *P2RX3*, was validated by both qRT-PCR analysis and western analysis. The *P2RX3* gene encodes a purinergic receptor which functions as a ligand-gated ion channel and mediates peripheral pain responses [27]. EtOH has been shown to epigenetically alter *P2RX3*, which may have downstream effects on the regulation of ion transport and may lead to reductions in hESC pluripotency [20]. We suggest it may also have a functional significance in alcohol-induced neuropathy. qRT-PCR analysis was performed to compare the relative fold changes of *P2RX3* expression in undifferentiated H1 hESC and hESC-derived NPC treated with EtOH. *P2RX3* in undifferentiated hESC was downregulated and conversely upregulated in NPC with increased EtOH concentration. (Fig 6A). We included 50 mM EtOH to examine if there is a dose dependency or any potential variations in response to different doses of EtOH. *CNTNAP4* and *P2RX3* genes are among upregulated genes that are upregulated by EtOH treatment. To examine if the alteration is due to EtOH’s effect we have concomitantly treated NPCs with EtOH and dihydromyricetin (DHM), a flavonoid component of herbal medicines that counters acute EtOH intoxication [28]. As shown in Fig 6B, NPCs treated with 20 mM EtOH showed induced level of *CNTNAP4* and *P2RX3* mRNA compared to untreated NPC. The induction was reduced by co-treatment of 1 μM DHM. Furthermore, undifferentiated H9 hESCs and H9 NPC cell lines were treated with 0, 20, and 50 mM EtOH for 48 hrs. From Western analysis, we have confirmed alcohol-induced downregulation of *P2RX3* in undifferentiated hESCs and upregulation in hESC-derived NPCs compared to beta-actin or p84 expression (Fig 6C). This result demonstrates *P2RX3* gene as one of molecular targets that are potentially deregulated by EtOH during neural differentiation process.

## Discussion

Various factors, signaling molecules, and other medias have been applied to differentiate ESCs into various neural precursor cells (NPCs). Some of them include: stromal-derived inducing cells, retinoic acid, bone morphogenetic protein, fibroblast growth factor receptors, and neurobasal medium [29]. ESCs can differentiate into the columnar neuroectodermal cells known as neural rosettes and can mimic *in vivo* neuroectodermal development in terms of timing and morphology [30]. Neural rosette cells can proliferate by self-renewal over a certain period of time and contain neural progenitor cells, which can further differentiate into more restricted progenitors leading to neuronal and glial cell lines[31, 32]. NPCs express high levels of neuroepithelial markers such as nestin, Pax6, Sox1, and maintain expression of Sox2 [33]. Differentiation potential of rosette cells diminishes with proliferation, occurring *in vivo* during neural development. Currently, there are many studies that focus on differentiation of ESCs for specific regions of neural cells as the nervous system consists of different regions.

Neural differentiation of ESC and neurogenesis pathway integrates complex epigenetic processes that require strict spatial and temporal cues. Major epigenetic mechanisms including DNA methylation, covalent histone posttranscriptional modifications, chromatin organization, and non-coding regulatory RNA play critical role in pluripotency maintenance and cell fate determination[34]. For instance, decreased DNA methylation activates neuronal genes such as Sox2 during ESC differentiation. More importantly, high levels of DNMT1 have been found to be crucial in DNA methylation during neural development. Furthermore, histone acetyl transferases and histone deacetylases are also known to be key players in regulation of ESC differentiation. Yet, the exact role of histone acetylation in embryonic development in terms of NSCs and brain development has not been clearly elucidated [35].

In relation to this study, epigenetic regulation of the neural transcriptome and the alcohol interference has recently studied and discussed widely. Resendiz et al. have elaborated on the alterations in neural stem cell development and on how alcohol leads to neurodevelopment deficits through neuroepigenetics [36]. As alcohol inhibits the differentiation of NSCs, cellular growth, migration, and cell viability are all negatively affected [37]. In specific, NSCs treated with EtOH exhibit cell cycle delays, reduced NSC proliferation and increased DNA fragmentation. In addition, EtOH are found to block the intrinsic hypermethylation of the cell cycle genes such as Adra1a, Tnf, Pik3r1, and Sh3bp2 during differentiation [38]. Moreover, the pluripotency genes Oct4, Sox2, and Nanog have demonstrated an EtOH-specific delay of down-regulation in NSC models [36].

Additionally, gene expression analysis of hESCs that differentiate into neural cells has facilitated in further defining the molecular mechanisms of neural development. Nestin and βIII-tubulin (TUBB3), which are cytoskeleton proteins, are marker proteins of neural stem cells and neurons respectively [39]. Nestin, expressed by neural progenitor cells, is specifically involved in the cytoskeleton remodeling and central/peripheral nervous system development. It has been found that nestin deficiency leads to embryonic lethality through the developmental defect of neural tubes with low NSCs. On the other hand, βIII-tubulin is an identifier for neurons that is also involved in neural development. In fact, mutations in the tubulin-encoding gene TUBB3 are found to yield a set of disease symptoms via microtubule dynamics in neurons [40]. Interestingly, it has been reported that there is a contrast in expression patterns of the two genes in the NPCs derived from the hESC H9 cell line: Nestin showed high expression while βIII-tubulin showed low expression, as well as random distribution[39]. It has also been noted that nestin is highly expressed during early stages of neural development but becomes downregulated in mature neurons whereas βIII-tubulin is expressed robustly in the later stages of the neurogenesis in both the cell soma and the neurite-like structures. Besides nestin and tubulin, there are other classic pluripotency markers such as Oct4, Sox2, ALP, and Nanog, as well as Sox1 and Pax6, which are upregulated during neural induction.

Our study was aimed to assess molecular effect of alcohol on the process of neural differentiation. Potential teratogenic effects of alcohol on fetal development have been documented. Especially studies have demonstrated deleterious effect of ethanol exposure on neuronal development in animal models and on the maintenance and differentiation of neuronal precursor cells derived from stem cells. Molecular and cellular effects of alcohol on neural stem cells and neural progenitors have been demonstrated [17, 20, 37, 38]. Several studies have shown that alcohol has a significant molecular effect on neural physiology. However, it has not been studied if alcohol exposure *in utero* has any molecular effect on the onset of the lineage specification of hESCs into neural precursors. Our recent study using hESCs demonstrated significant ethanol-induced molecular alterations in hESCs, especially through epigenetic mechanism [20].

In this study, we have generated neural stem cells from pluripotent hESCs and examined global transcriptomic signature changes affected by ethanol treatment (Gene Expression Omnibus under GSE56906), which will provide scientific insight on potential molecular effects of fetal alcohol exposure on neural differentiation of early embryo development [24]. In particular, we have identified and verified several candidate genes that alcohol could interfere in neural stem cell regulation. Major molecular pathways in NPCs affected by EtOH have been demonstrated as associated with alcohol exposure, such as JAK-STAT signaling pathway [41–43], neuroactive ligand-receptor interaction [20, 44, 45]and Toll-like receptor (TLR) signaling pathway [46, 47]. Among verified genes, we have further examined EtOH’s effect on *CNTNAP4* and *P2RX3*. *CNTNAP4* is Contactin Associated Protein-Like 4 and belongs to the neurexin family, members of which function in the vertebrate nervous system as cell adhesion molecules and receptors. It is a presynaptic protein involved in both dopaminergic synaptic transmission and GABAergic system, thereby participating in the structural maturation of inhibitory interneuron synapses. Involved in the dopaminergic synaptic transmission by attenuating dopamine release through a presynaptic mechanism. Studies have demonstrated a potential association of *CNTNAP4* autism [48–50] or alcohol dependence. *P2RX3*, Purinergic Receptor P2X, Ligand Gated Ion Channel 3, belongs to the family of purinoceptors for ATP. It functions as a ligand-gated ion channel and may transduce ATP-evoked nociceptor activation [51, 52]. It needs to be demonstrated how these genes are dysregulated in NPCs upon EtOH exposure and what are the significance of their dysregulation in mediating EtOH’s effect on NPC development. Further functional assessment using our model will help us delineate molecular mechanisms and understand cellular consequence of alcohol’s effect on neural stem cell regulation.

## Supporting Information

S1 Fig Quantitative assessment of neural differentiation. **A.** The level of neural makers (Nestin, Tuj1 and Musashi1) was assessed by qRT-PCR in rosette and NPCs and compared to undifferentiated hESCs. **B.** The effect of 20 mM EtOH treatment on neural markers (Nestin, Tuj1 and Musashi1) in neural rosette and NPCs was determined by qRT-PCR assay. **C**. The level of pluripotent markers, Oct4 and Sox2, was assessed in undifferentiated hESC (Und) and NPCs without (0 mM) or with (20 mM) EtOH treatment. Bars are mean ± SEM from triplicates; the asterisk denotes significant (*p*<0.05) difference from control (one-way ANOVA).
(TIFF)
