# Supplementary figures and images for: Alcohol-Induced Molecular Dysregulation in Human Embryonic Stem Cell-Derived Neural Precursor Cells
Source: PLoS One. 2016 Sep 28;11(9):e0163812. doi: 10.1371/journal.pone.0163812 (PMC5040434; doi:10.1371/journal.pone.0163812)

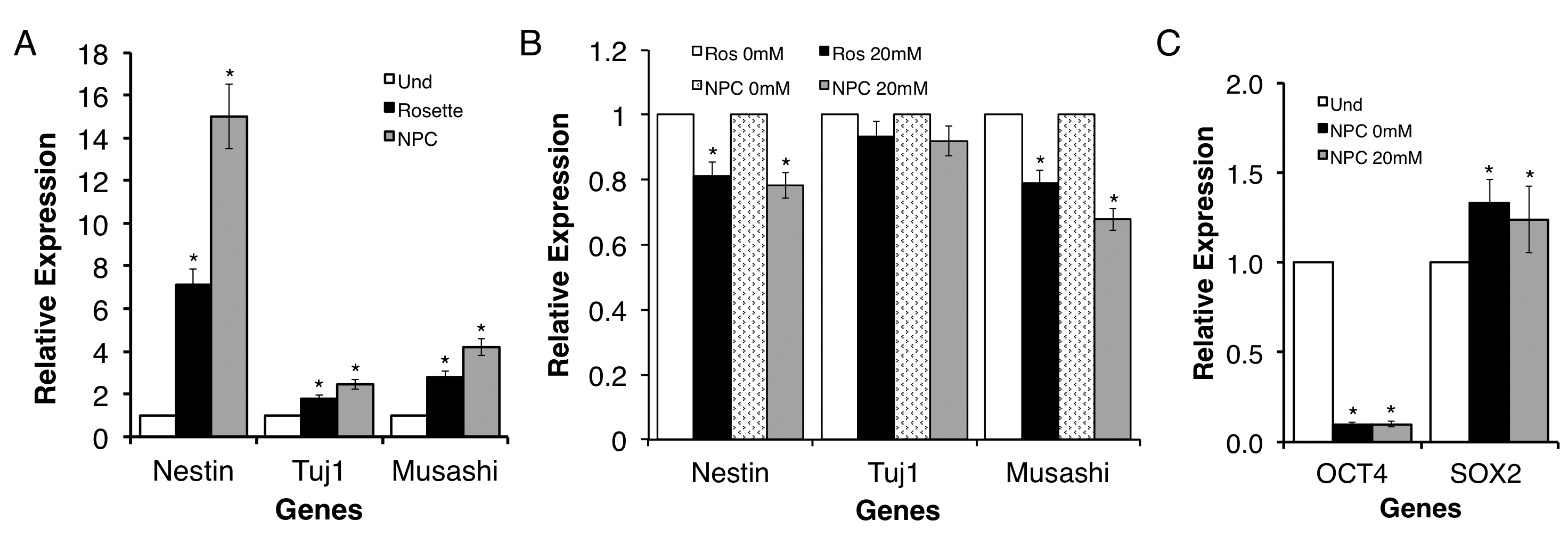

Supplement: S1 Fig — A. The level of neural makers (Nestin, Tuj1 and Musashi1) was assessed by qRT-PCR in rosette and NPCs and compared to undifferentiated hESCs. B. The effect of 20 mM EtOH treatment on neural markers (Nestin, Tuj1 and Musashi1) in neural rosette and NPCs was determined by qRT-PCR assay. C. The level of pluripotent markers, Oct4 and Sox2, was assessed in undifferentiated hESC (Und) and NPCs without (0 mM) or with (20 mM) EtOH treatment. Bars are mean ± SEM from triplicates; the asterisk denotes significant (p<0.05) difference from control (one-way ANOVA). (TIFF) [file pone.0163812.s001.tiff]
